# The Use of Information Communication Technologies Among Children With Autism Spectrum Disorders: Descriptive Qualitative Study

**DOI:** 10.2196/12176

**Published:** 2019-09-27

**Authors:** Theoneste Ntalindwa, Tanjir Rashid Soron, Mathias Nduwingoma, Evariste Karangwa, Rebecca White

**Affiliations:** 1 School of Education University of Rwanda Rukara Rwanda; 2 Telepsychiatry Research and Innovation Network Ltd Dhaka Bangladesh; 3 School of Nursing and Midwifery University of Illinois at Chicago Kigali Rwanda

**Keywords:** autism spectrum disorders, information communication technologies, inclusive education

## Abstract

**Background:**

The prevalence of Autism Spectrum Disorder (ASD) appears to be increasing globally due to the complex interaction of multiple biopsychosocial and environmental factors. Mobile phones, tablets, and other electronic gadgets have transformed our means of communication, and have also changed both healthcare and how we learn. These technological enhancements may have a positive impact on the lives of children, but there is currently a global scarcity of information on how information technology influences the education of children with ASD.

**Objective:**

This study was conducted in Rwandan schools and communities, and aimed to understand the perceptions of students with ASD, their parents, and their teachers, on the use of Information and Communication Technology (ICT) in the education of those with ASD.

**Methods:**

This qualitative descriptive study was conducted from December 2017 to July 2018. Researchers conducted four focus group discussions (FGDs) with 54 participants from different backgrounds: teachers, parents, and students with ASD. Each of the FGDs took approximately two and a half hours. A predefined set of open-ended questions were selected to discover people’s perceptions regarding assistive technologies used in ASD, their effectiveness, the scope of using them in their context, and upcoming challenges during implementation. The interviews were recorded, transcribed, and analyzed.

**Results:**

The findings of the study revealed seven key themes: (1) the use of ICT for the education of children with ASD; (2) existing augmentative facilities for learning; (3) current patterns of use of ICT in education; (4) preferred areas of learning for ASD students; (5) integration of ICT into educational programs; (6) areas of interest outside the classroom; and (7) future opportunities and challenges in Rwanda. We found most of the study participants assumed that appropriate technology and related innovations might solve the challenges faced by learners with ASD in classrooms. Moreover, they thought that children with ASD more so enjoyed watching television, playing digital games, and drawing objects using gadgets than interacting with people or playing with other children.

**Conclusions:**

The use of various low-cost technical devices can aid with teaching and the education of children with autism in Rwanda. However, this area requires further research to discover the impact ICT can have on the education of children with ASD, so this study may become a starting point for further research in the area.

## Introduction

Like any other child, those children with Autism have an equal right to education and all other basic rights [1]. However, students with Autism Spectrum Disorder (ASD) face several challenges during their education in both developed and low- and middle-income countries (LMICs) [2,3]. Despite the Ministry of Education’s policy on the education of children with disabilities and other special needs in, which has been active since January 2019 [4,5], schools continue to demand comprehensive guidelines on effective teaching and education, standard infrastructural facilities, resources, professional educators, and support services. Besides that, the lack of logistical support seems to be the major source of children with ASD’s generalized poor performance in numeracy, literacy and comprehension skills [6]. To overcome the challenges in schools, Rwanda is promoting the use of ICT at all levels through multiple initiatives that include the One Laptop Per Child Project at basic education levels, and loan schemes for students in higher learning institutions [7,8]. These interventions have increased the ratio of number of computers per user [9] and reduced the gap of access to Information and Communication Technology (ICT) in urban and rural populations [10]. Adoption of simulation-based multimedia technologies [11] have improved basic computer skills (eg word processing and spreadsheet applications) in ordinary Rwandan schools, but not necessarily in the country’s inclusive and special schools [12].

Information communication technologies and modern education techniques can assist with the education of a person with disabilities by mitigating their challenges in different domains [13-17]. The use of assistive technology to enable those with ASD in their communities has been emphasized by The United Nations (UN) [18,19], and it has been assumed that the use of assistive technologies will improve the performance of children with ASD at school [20]. Assistive technologies benefit people with ASD by improving their ability to interact socially [21] and their self-engagement [22]. Moreover, ensuring inclusive and equitable education and promoting lifelong learning for all is also part of the UN’s national comprehensive education strategy, which contributes to achieving Sustainable Development Goal-4 [23]. However, Rwanda’s education programs are still limited in many ways by limited research evidence on autism [24], specifically on the use of adapted technologies geared towards improving learning competency. Thus, this study was conducted with the deliberate aim of addressing this gap.

## Methods

This qualitative descriptive study was conducted from December 2017 to July 2018, with the objective of understanding how ICT can support the education of children with ASD. The educational institutions chosen for focus group discussions were selected based on data from the National Commission of Persons with Disabilities and the Ministry of Education in Rwanda [25]. Researchers used structured interviews following the guidelines of qualitative research [26]. Participants were interviewed to find out their views and opinions on the effectiveness and use of ICT in an inclusive classroom setting. Children with ASD, their teachers and their parents participated in focus group discussions (FGDs). The FGDs were conducted in three randomly selected districts from two different provinces, and the study sites included: Groupe Scolaire Jabana (Gasabo District), Heroes Day Care Center (Kicukiro District), Autisme Rwanda within Kigali City, and the College des Amies de la Paix du Christ Roix (APAX) Janja (Gakenke District) located in the northern province of Rwanda. Teachers who participated in this study taught a variety of subjects, including mathematics, ICT, creative performance, geography, English, tailoring, chemistry, history, biology, and Kinyarwanda. Each school was represented by an equal number of teachers, parents, and children with ASD, with five teachers (N=5), three parents (N=3) and four children with ASD (N=4). Six children with standard intellectual capacity were selected from two inclusive schools which are implementing the competence-based curriculum, and they were the Groupe Scolaire Jaban (N=3) and the College des Amies de la Paix du Christ Roix (APAX) Janja (N=3).

Within the parents’ group there were four fathers and eight mothers. Children with standard intellectual capacity from each school participated solely in observational sessions. Table 1 below describes 54 participants who were systematically divided into four FGDs based on the location of the four schools selected.

**Table 1:** Number of participants in focus group discussion (N=54).

Participants	Groupe Scolaire Jabana	Heroes Day Care Center	Autism center (Autisme Rwanda)	College des Amies de la Paix du Christ Roix Janja	Total
Teachers	5	5	5	5	20
Parents	3	3	3	3	12
Students with ASD^a^	4	4	4	4	16
Children with average intelligence	3	0	0	3	6
Total	15	12	12	15	54

^a^ASD: Autism Spectrum Disorder

The mobile apps chosen to support the education of children with ASD, such as *Mental Math Expert* [27] and *Milk Hunt* [28], were randomly selected from the internet and installed on laptops and smartphones that were used by both children with ASD and those with standard intellectual capacity to observe and compare their ability to use these digital devices. Each of the FGDs took approximately two and a half hours, and three observational sessions were performed to observe the ability of children with ASD to use ICT tools.

The answers from the interviews and results from the observational sessions were analyzed and transcribed using Word (Microsoft, Redmond, Washington). To clean the data, a coding scheme was developed with codes serving as groups of teachers, parents, and children with ASD in light of their knowledge, experiences, and perspectives. We refer to teachers, parents, and children using the following notation: T1 refers to teacher 1, P1 refers to parent 1, C1 refers to child 1, and so on.

The data were collected by recognized researchers from the University of Rwanda who have been trained on qualitative data collection, and the research project passed through the collegial ethical process. To maintain ethical standards, the researchers took formal informed consent from parents and teachers using approved consent form (Multimedia Appendix 1) and also used few prefixed questions to facilitate the Focus Group Discussion (Multimedia Appendix 2).

## Results

### Overview

The findings of the study revealed seven key themes: 1) the use of ICT for the education of children with ASD; (2) existing augmentative facilities for learning; (3) current patterns of use of ICT in education; (4) preferred areas of learning for ASD students; (5) integration of ICT into educational programs; (6) areas of interest outside the classroom; and (7) future opportunities and challenges in Rwanda.

### The Use of ICT for the Education of Children with Autism Spectrum Disorder

Despite the financial barriers and lack of robust funding, schools and parents were provided digital tools for children with ASD. Rwandan financial leadership in education encouraged promoting facilities and increasing the capacity of ICT at the school level:

I encourage the government of Rwanda to provide technical support to develop their learning abilities as well as including them into general education system.

A teacher from Groupe Scolaire Jabana

However, technological innovations such as educational software applications and videos need to be culturally relevant to both support students with ASD and improve their attention when they are studying. A teacher from APAX-Janja said

Special Software Applications and videos are essential in the class to help students paying attention as most of my students with ASD enjoy watching the video. When they are left alone watching, they are more focused and finally imitate what they have seen through media.

The advanced technology integrated well into teaching children with ASD, and the provision of an abundance of new tools to educators to use in their instruction helped them be more efficient. This was especially evident when the emphasis was on symbolic notation and how children might learn from them, as supported by most of the teachers during the interview.

### Existing Augmentative Facilities for Learning

The teachers from the four schools reported that only a few of them had a computer or tablet with special software applications for a person with ASD, such as games like *Matchit* and *Number Run* that are designed to work on basic mathematics skills. All schools and centers have the same educational barriers related to a lack of qualified staff that can support students with ASD, which are compounded by facility limitations including classrooms, dormitories, and teaching materials which are not adapted to them. Thus, an increase of facilities to support the education of children with ASD is recommended by school educators.

### Current Patterns of Use of Information and Communication Technology in Education

It has been thought that cognitive software applications can have a positive effect on the education of a person with Autism, with one of the interviewed teachers saying:

I find the students with ASD and related disabilities are more interested in the use of mobile telephones and tablets which we are using to improve their communication at the school. They can search the application from tablet by themselves and start using it.

a teacher from the Autism Center in Rwanda

Despite these positive intentions, teachers reported difficulties in the use of new technologies with children with ASD. Teachers at Heroes Day Care Center said:

What we do here is that we try all means to get some tools to support our children, but all of them are in English or French language which is a new language and difficult for them to learn as they have difficulty in communicating in our local language.

More teachers and parents suggested an increase of use of ICT tools with specific software applications installed, as well as training in teaching of and caring for children with ASD in an inclusive environment.

### Preferred Areas of Learning for Autism Spectrum Disorder Students

In response to the question of interests of students with ASD, teachers said that the students were interested in subjects which involved vision and hearing. They said that many students with ASD liked the subjects that involved drawing and creative performance (eg, singing and dancing), while a few of them had unknown interests. This was supported by a teacher at Groupe Scolaire Jabana who said:

in my class, those children most like geometry, but when I teach algebra, they do not follow, I sometimes find them drawing the faces of their colleagues and teachers. They are also able to see some objects which other children considered as having normal intellectual capacity are not interested in.

From the researchers’ observations, students who participated in the observational session demonstrated different individual capabilities, such as drawing the objects that they saw, reproducing specific images, and memorizing songs and voices that they heard.

### Integration of Information and Communications Technology into Educational Programs

Parents stated that their children liked to use digital devices and recommended the provision of access to particular ICT tools. One father of a child with ASD said:

My son is very good at playing games on my smartphone, and he is the one who usually open a TV for me and forces me to watch a channel of cartoons. If the government provide ICT tools for children like mine at school, it will be better.

Teachers and parents thought that the integration of ICT and the provision of cost-effective assistive technology devices would be helpful. A teacher from APAX Janja said:

If we can provide the low-cost tablet or smartphone with special applications for each of the children customized with their interest, and provide the teaching with that, I am confident that they will be more successful in their specific domains.

This statement was supported by teachers who used ICT tools to support teaching their children in class, with all of them saying that ICT could improve both teaching and learning activities for children with ASD in inclusive settings.

### Areas of Interest Outside the Classroom

Parents from urban areas said that their children liked watching television and playing digital games, while those from rural areas said that their children liked drawing objects they saw around and imitating the sounds and voices of animals and people. The parents also said that their children demonstrated a deficit in socialization with others in their families, but they were seen engaging in different activities. Another father of children with ASD said:

I have never seen my son talking about his interests or get engaged in activities I gave him. It took me a long time in observing him and I surprisingly saw him get engaged in different activities while I thought he is not able to do anything.

This testimony was also supported by a Teacher at APAX Janja who said:

I remember when I was doing my academic research, I met one man who was diagnosed as having Autism in Kigali who can imitate most of the voices of animals like, dog, cow, bird, lion, and others. If digital tools like Television is available to young children, they can imitate what they are watching.

The parents also said that their children could perform some house activities independently. From observations in the workshop room, children with ASD demonstrated a good level of skill in tailoring sweaters and performing other creative activities.

### Future Opportunities and Challenges in Rwanda

The disruptive behavior of students in the inclusive classroom may be a barrier to the effective implementation of the proposed competence-based curriculum. This was supported by teachers who said:

The behaviors of children are very challenging, and it is not easy to teach them with others regardless of the policy of the government emphasizing their inclusion in schools. It will only happen if we have two or three teachers in one classroom to support them.

In this study, most teachers in the schools and centers suggested that the presence of two teachers in an inclusive classroom could help with overcoming the challenging behaviors of children with ASD.

Parents also reported a lack of awareness of ASD as a factor which supported the stigmatization of their autistic children among different families, and it also seemed to encourage them to drop out of the schools.

My family failed to accept the behavior of my daughter, and the cost of caring for her. They always ask me where I got her and I am afraid of her future when I will not be alive. But I believe in that with the help of ICT like Television broadcasts and Radio talks, they can change their beliefs within the time.

A mother of an ASD daughter

Using digital technologies to increase awareness of the disorder is thought to be a solution for reducing stigma among families.

In addition, the participants also said that a combination of a lack of materials, qualified teachers, awareness, and teachers’ motivation was a significant challenge to including children with ASD into the Rwandan education system.

## Discussion

### Primary Findings

This study revealed the use of ICT was acceptable among both teachers and parents of children with Autism. The triangulation of themes developed in the findings shows that the participants in the study thought that ICT had the potential to improve the education of children with ASD in class regardless of the problem of an insufficient number of tools that have been developed [29]. The use of assistive technologies has previously proven to have the potential to improve the education of children with ASD in Rwanda and beyond, as supported by multiple studies [30-32]. Despite the importance of ICT in supporting the education sector, the lack of facilities are a significant challenge in enhancing the learning of students with autism in mainstream schools [33-35]. This results from the multidimensional constraints of culturally valid and acceptable educational curricula, inadequate training for teachers, and a lack of strategies for teachers to improve their methodologies when they are teaching in inclusive classroom settings [36-37].

These findings are supported by previous studies [38-40] and by the recommendations of the educators who participated in this study. Children with ASD perform better when an emphasis is made on the augmentation of digital technologies, cognitive software applications, and the senses of children with ASD (ie, hearing and vision) [41-43]. This is supported by our observational findings, which indicated an improvement in the ability of the child participants to use cognitive software applications installed on the computers and smartphones provided to them.

To support previous research [44], the parents who participated in the FGDs stated that their children with ASD were motivated to engage in different activities at home, and the role of parents and teachers should be to guide and supervise rather than to compel them to work on a task. The interviewed teachers suggested improving the competency-based curriculum by focusing on mental disorders such as ASD, as supported by varying studies [45,46]. The parents and teachers also reported that some children were not attending school due to stigmatization [47]. However, the findings of this study suggest that increasing awareness of ASD by using varying means, including ICT, could help to overcome the stigma against people with ASD that has been associated with social and cultural issues [48]. This is supported by Ehsan et al [49], who found a lack of contextualized studies on ASD in less developed countries while the number of people with ASD continues to increase.

As a developing country, Rwanda has a lack of digital infrastructure that is a challenge and can lead to reduced support for people with ASD [49]. This supports studies which prove that digital technologies, when used effectively, can deliver foundational educational content to any learner so they can respond to the needs of people with ASD from different geographical contexts [40,50]. In addition to other research [51,52], this study recommends further exploration into LMICs such as Sub-Saharan Africa.

### Limitations

This study used only focus group discussions and observational sessions, which could have made the results prone to selection biases, resulted in inequal engagement of the participants, and could have been influenced by the person who conducted the FGDs. However, the participants were selected randomly based on data from a recognized National Commission of Persons with Disabilities in Rwanda, which minimizes the bias, and careful monitoring was done to ensure everyone participated and shared their opinions during the FGDs. Our results may be conservative and underestimate the full education and health benefits of children with ASD, as in the selected schools only two are currently implementing the competence-based curriculum while others are using a special curriculum to improve the lives of children with ASD. In addition, among the five selected schools only one was from a rural district of Rwanda, which may have resulted in a deficit of required study information in the rural area. Third, we based our study only on children with ASD enrolled in the school system and in caring centres, but there are others who do not attend schools or specialized centres because their education is expensive. Finally, parents who participated in the study were only those who had children enrolled in the school system or centres, but some research has found that some parents do not allow their children to attend school due to the stigma associated with ASD.

We did not include teaching models and methodologies, as this study only focused on the use of ICT for teaching children with ASD. Our results cannot replace the evidence of other existing methods and tools which are used to support the education of children with ASD, as this should be guided by different studies from multiple domains accompanied by robust independent assessment.

### Conclusions

Based on these findings, the integration of ICT into the Rwandan educational system is essential to support and build the competency of children with ASD. Increasing societal awareness of ASD and enhancing the motivation of Rwandan teachers would help to reduce the stigma of ASD for families and within communities. This study will help future researchers in this domain in looking at the use of ICT for people with ASD, with program implementation, and completing a similar evaluation in a larger population sample. Recommendations also include new education-based ICT research in resource-limited settings with the general population as compared to ICT-based education within the ASD student population.

## Appendix

Multimedia Appendix 1 Consent form.

Multimedia Appendix 2 Predefined question.

## Abbreviations

APAX: College des Amies de la Paix du Christ Roix
ASD: Autism Spectrum Disorder
FGD: focus group discussion
ICT: Information and Communication Technology
LMIC: low- and middle-income countries
UN: United Nations
