# Supplementary material for: The Use of Information Communication Technologies Among Children With Autism Spectrum Disorders: Descriptive Qualitative Study
Source: JMIR Pediatr Parent. 2019 Sep 27;2(2):e12176. doi: 10.2196/12176 (PMC6789423; doi:10.2196/12176)
Supplement: Multimedia Appendix 1 [file pediatrics_v2i2e12176_app1.pdf]

Appendix 1. Informed Consent Form for teachers/Parents

UNIVERSITY of
COLLEGE OF EDUCATION

Informed Consent Form for teachers/Parents

This informed consent form is for teachers of children participating in the research study titled: "**Use of Information Communication Technologies among children with Autism Spectrum Disorders; A descriptive qualitative study**"

The study is conducted by Théoneste Ntalindwa (PhD candidate at University of Rwanda, Rwanda), Nduwingoma Mathias, Karangwa Evariste and Tanjir Rashid Soron (Neuro Developmental Disabilities Protection Trust, Bangladesh).

This Informed Consent Form has two parts:

- Information Sheet (to share information about the study with you)
- Certificate of Consent (for signatures if you agree that your child may participate)

Part 1: Information Sheet

Introduction

We are doing research to enable people with Autism Spectrum Disorders to be included in basic education system in Rwanda. In short, the study is about finding out how can be useful to support teaching and learning in Rwandan primary education schools.

In our research, they will be open discussion about the ICT to improve teaching and learning of children with ASD. The focus group discussion will take place at school address.

To do this we ask you as a teacher / parent for permission. After reading about the study below, and if you agree, then the next thing we will do is ask you for their agreement as well, before the session.

There may be some words that you do not understand. In that case, please feel free to either contact us via e-mail or the principal at the school, who can send us all your questions via e-mail so we can answer it before you sign the certificate of consent.

Voluntary participation

You do not have to agree that you can participate in the study. You can choose to say no and any services that you receive at the school will not change. We know that the decision can be difficult. You can ask as many questions as you like, and we take the time to answer them via the school manager. You do not have to answer any question or take part in the focus group discussion if you feel uncomfortable doing so.

Procedure

The study will be done at the <<**Name of the school>>>.** You will participate in one session of about one hour and half in focus group discussion. The interviews will be recorded.

The questions that are likely to be asked during the focus group discussion will be related to the main research question: How useful is ICT to support education of children with ASD learning in Rwanda?

Benefits

There will be no immediate and direct benefit to you or your school, but your participation is likely to help us find out more about how the ICT can be made more accessible and support inclusion of person with neuro-developmental disabilities like ASD in education system with others normal students. We hope that the results will help integration of ICT in your teaching profession and enabling your child to perform like others at the school as well as improving communication between you and your children.

Reimbursements

You will not be provided with any payment to take part in the research. However, the findings of research will be available to you for free.

Confidentiality

We as researchers will not share information about you. However, because something out of the ordinary is being done through research in your community, it will draw attention. If you participate, you may be asked questions by other people in the community. We cannot guarantee confidentiality, but it is our belief that the nature of the research question is not very sensitive or personal.

Sharing of Research Findings

We will share what we have learnt with the participants and the principal. Nothing that your child will tell us during the sessions will be attributed to him/her by name. A written report will also be given to the participants, which they can share with their community. We will also publish the results in order that other interested people may learn from our research.

Right to refuse or withdraw

You may choose not to participate in this study. Choosing to participate or not will not affect either your own future at the school in any way. You still have all the benefits that would otherwise be available at this school. You stop participating in the discussion at any time that you wish without either of you losing any of your rights.

Who to Contact

If you have any questions you may ask them now or later, even after the study. If you wish to ask questions, you may contact either the principal or Théoneste Ntalindwa, ntatheos@yahoo.co.uk , Telephone: 0788884594. Mathias Nduwingoma ndumathias2001@yahoo.com, Telephone: 0788897814. Evariste Karwanga karangwa81@ymail.com, Telephone: 0785489767.

Part 2: Certificate of Consent

I as a teacher have been asked to give consent to participate in this research study, which will involve observation and a brief interview. I have read the foregoing information, or it has been read to me. I have had the opportunity to ask questions about it and any questions that I have asked to have been answered to my satisfaction. I consent voluntarily to participate in this study.

Print Name of Teacher ___________________________________

Signature of Teacher____________________________________

Date __________________________

Day/Month/Year
