# Supplementary material for: The Use of Information Communication Technologies Among Children With Autism Spectrum Disorders: Descriptive Qualitative Study
Source: JMIR Pediatr Parent. 2019 Sep 27;2(2):e12176. doi: 10.2196/12176 (PMC6789423; doi:10.2196/12176)
Supplement: Multimedia Appendix 2 [file pediatrics_v2i2e12176_app2.pdf]

Appendix 2. Interview guide and Characteristics of a Person with Autism Spectrum Disorder

1. **Characteristics of a Person with Autism Spectrum Disorder**

Autism is characterized by marked difficulties in behavior, social interaction, communication and sensory sensitivities.

1. **Behavioral**

People on the spectrum may exhibit unusual behavior due to the difficulties they have responding to their environment. These behaviors may include:

- unusually intense or focused interests
- stereotyped and repetitive body movements such as hand flapping and spinning
- repetitive use of objects such as repeatedly switching lights on and off or lining up toys.
- insistence on sticking to routines such travelling the same route home each day and doing things in exactly the same order every time
- sensory sensitivities including avoidance of everyday sounds and textures such as hair dryers, vacuum cleaners and sand

1. **Social interaction**

Their difficulties with social interaction may manifest in the following ways:

- limited use and understanding of non-verbal communication such as eye gaze, facial expression and gesture
- difficulties forming and sustaining friendships
- lack of seeking to share enjoyment, interests and activities with other people
- difficulties with social and emotional responsiveness

2. **Communication**

There are some people with autism who speak fluently, others who are speech impaired to varying degrees and others still, who are unable to speak at all. Impaired communication is characterized by:

- delayed language development
- difficulties initiating and sustaining conversations
- stereotyped and repetitive use of language such as repeating phrases from television

2. **Interview guide**

a. **questions for teachers .**

1. As I come from describing the characteristics of children with ASD, do think you have some children in your class?
2. I am conducting research into basic education. Inn which level you you are teachning?
3. Which subject are you teaching?
4. Which subject your students with ASD like most?
5. What are the interests of children with ASD in class?
6. My research interest is ICT to support education of children with ASD in schools.
   Do you have some ICT gadget to support your teaching activities?
7. Do you have any special tool designed only to help those learners with ASD?
8. Do the students have access to ICT tools?
9. Which topics the learners mostly like?
10. Do you think ICT can improve performance in these topics?
11. How ICT can be used to teach these children?
12. What do see as the biggest obstacle to your learners with ASD developing their interests?
13. In your teaching, what do you suggest that can improve your teaching activity?
14. What is your message to the government regarding education of children with ASD?

b. **questions for parents**

1. As I come from describing the characteristics of children with ASD, do think you have some children in your class?
2. How did find the school for your children the ASD is not known by every school?
3. Did you know about Autism
4. How your children behave out of the schools
5. Do you have any digital tool at your home such as TV, smartphone, Radio, etc. ?
6. How often you children use those tools
7. What are behavior changes you see from the students after using digital tool?
8. What the interest of those children who do not have access to the smartphone?

9. Did you see you children engaged in some activities at home?
10. How do you think ICT can contribute to educate your children?
11. What are difficulties you observe the children face when they are using ICT tools?
12. In which area should we emphasize whewn selecting ICT tools for these children?
13. What do you see as the biggest obstacle to your children with ASD developing his interests?
14. What is your message to the government regarding education of children with ASD?
15. What is your message to the educators?
16. What is your message to the family of children with ASD?

c. Interview guide questions for shool managers

1. As I come from describing the characteristics of children with ASD, do think you have some children in your class?
2. I am conducting research into basic education. Are you teaching in which levels?
3. Are your class inclusive? This means all students with and without ASD are in the same class.
4. Which subject your students with ASD like most?
5. What are the interest of children with ASD in school activites?
6. My research interest is ICT to support education of children with ASD in schools. Do you use ICT as a tool in your teaching activities?
7. Do you have any special ICT designed only to help those learners with ASD?
8. Do the students have access to ICT tools?
9. What are facilities you have at this school?
10. Which topics the learners mostly like?
11. Do you think ICT can improve performance in these topics?
12. How ICT can be used to teach these children?
13. What do see as the biggest obstacle to your learners with ASD developing their interests?
14. How the policy of inclusion in education is implemented at your school?
15. What is your message to the government regarding education of children with ASD?
